# Angiogenesis enhanced by treatment damage to hepatocellular carcinoma through the release of GDF15

**DOI:** 10.1002/cam4.1330

**Published:** 2018-01-31

**Authors:** Gang Dong, Qiong‐Dan Zheng, Min Ma, Si‐Fan Wu, Rui Zhang, Rong‐Rong Yao, Yin‐Ying Dong, Hui Ma, Dong‐Mei Gao, Sheng‐Long Ye, Jie‐Feng Cui, Zheng‐Gang Ren, Rong‐Xin Chen

**Affiliations:** ^1^ Liver Cancer Institute Zhongshan Hospital Fudan University and Key Laboratory of Carcinogenesis and Cancer Invasion, Ministry of Education Shanghai China

**Keywords:** Angiogenesis, GDF15, hepatocellular carcinoma, thalidomide, transarterial chemoembolization

## Abstract

Transarterial chemoembolization (TACE) is the standard treatment for unresectable hepatocellular carcinoma (HCC). Hypoxia‐induced angiogenesis by TACE is linked to treatment failure; however, whether the chemotherapeutic damage of TACE to HCC could increase tumor angiogenesis has not been explored. The molecular effects of chemotherapy‐damaged HCC cells on the neo‐angiogenesis were investigated in vitro and in vivo. The expression of growth differentiation factor 15 (GDF15) was significantly upregulated in HCC cells exposed to chemotherapeutic agents. GDF15 from chemotherapy‐damaged HCC cells promoted the in vitro proliferation, migration, and tube formation of endothelial cells. The pro‐angiogenic effect of GDF15 was through the activation of Src and its downstream AKT, MAPK, and NF‐κB signaling, which was blocked by thalidomide. The use of thalidomide significantly attenuated the in vivo chemotherapy‐damaged HCC cells‐promoted angiogenesis in nude mice. In conclusion, the chemotherapeutic damage in TACE to HCC could promote tumor angiogenesis via the increased release of GDF15. Thalidomide could reverse these pro‐angiogenic effects.

## Introduction

Hepatocellular carcinoma (HCC) is one of the most common malignant cancers in the world 1. Most of HCC patients are diagnosed at intermediate‐advanced stage and ineligible for radical therapies. Transarterial chemoembolization (TACE) is effective for unresectable HCC and provides the modest survival benefit for patients due to its noncurative procedure 2. Among the factors potentially interfering with its effectiveness, a presumed angiogenic reaction due to hypoxic induced by TACE is well known. For example, the increased angiogenic factor of vascular endothelial growth factor (VEGF) following TACE is associated with treatment efficacy and survival of patients 3. However, no improvement in tumor response or prognosis of patients was observed in HCC patients who received TACE and bevacizumab (a monoclonal antibody against VEGF), suggesting the complex neo‐angiogenic mechanisms after TACE 4, 5. As TACE not only exploits the embolization to induce ischemia but also combines the arterial infusion of chemotherapeutic drugs to produce the cellular damage, whether the chemotherapeutic damage of TACE to HCC could stimulate tumor angiogenesis has not been fully explored.

The phenomenon of neo‐angiogenesis is prevalent in sustaining tumor initiation and progression 6. The angiogenic rebound following TACE is linked to tumor progression of residual HCC, leading to treatment failure or refractoriness. Although the exact mechanisms of angiogenesis following TACE remain unclear, the hypoxia‐induced VEGF by TACE is the most frequently reported angiogenic factor responsible for tumor survival and regrowth after TACE 6, whereas the pro‐angiogenic effects of chemotherapy are neglected in previous studies.

Growth differentiation factor 15 (GDF15) is a multifunctional protein produced under various stress conditions 7. Carrillo‐García et al. 8 showed that GDF15 regulated the migration and proliferation of neural precursor by promoting EGFR signaling. Considering the relationship of EGFR with angiogenesis, we hypothesized that GDF15 secreted from chemotherapy damage to tumor cells could promote neo‐angiogenesis after TACE treatment, eventually leading to the residual tumor progression.

Here, we demonstrated that (1) GDF15 was significantly increased in chemotherapy‐damage to HCC cells; (2) GDF15 from chemotherapy‐damaged HCC cells promoted the in vitro proliferation, migration and tube formation of endothelial cells; (3) the pro‐angiogenic effect of GDF15 was through the activation of Src and its downstream AKT, MAPK and NF‐κB signaling, which was blocked by thalidomide; (4) thalidomide significantly attenuated the in vivo chemotherapy‐damaged HCC cells‐promoted angiogenesis.

From a new perspective of tumor angiogenesis enhanced by chemotherapy‐damage to HCC, we provide a novel insight into the angiogenic rebound following TACE and propose thalidomide as a potential drug to target this pro‐angiogenic process.

## Materials and Methods

### Reagents and antibodies

Cisplatin, doxorubicin, and thalidomide were obtained from Sigma‐Aldrich (St. Louis, MO). Recombinant human GDF15 was purchased from R&D Systems (Minneapolis, MN). Polyclonal rabbit anti‐human ERK1/2 antibody, polyclonal rabbit anti‐human p38 MAPK antibody, monoclonal rabbit anti‐human JNK antibody, monoclonal rabbit anti‐human P‐JNK antibody, polyclonal goat anti‐human GDF15 antibody and polyclonal rabbit anti‐human NF‐κB antibody (Abcam, Cambridge, UK), polyclonal rabbit anti‐human P‐ERK1/2 antibody (Thermo Fisher Scientific, Waltham, MA, USA), monoclonal mouse anti‐human P‐P38MAPK antibody, monoclonal rabbit anti‐human Akt antibody, monoclonal rabbit anti‐human P‐Akt antibody, polyclonal rabbit anti‐human Src antibody, polyclonal rabbit anti‐human p‐Src antibody, and monoclonal rabbit anti‐human p‐NF‐κB antibody (Cell Signaling Technology, Beverly, MA) were used for Western blots. Monoclonal rabbit anti‐human CD31 antibody and polyclonal rabbit anti‐human paxillin antibody (Abcam, Cambridge, UK) were used for immunohistochemical analysis and immunofluorescence, respectively. Src inhibitor Saracatinib was purchased from Selleckchem (Houston, TX).

### Cell culture

The human umbilical vein cell line EA.hy926 (ATCC, Manassas, VA, USA), HCC cell lines HepG2 (ATCC, Manassas, VA, USA), and Huh7 (obtained from the Japanese Cancer Research Resources Bank, Tokyo, Japan) were cultured in the Dulbecco's modified Eagle's medium (DMEM) supplemented with 10% fetal bovine serum and 1% penicillin–streptomycin. All cells were incubated in humidified atmosphere of 5% CO_2_ at 37°C.

To mimic chemotherapy damage of TACE to HCC, HepG2 and Huh7 cells were exposed to cisplatin or doxorubicin for 24 h.

To collect the conditioned medium (CM) from chemotherapy‐damaged HCC cells, Huh7 cells with 80% confluence were treated by cisplatin (6.9 μg/mL) for 24 h, then digested, seeded into the six‐well plate (10^6^/well), and incubated for 24 h with fresh DMEM. Fresh DMEM incubated Huh7 cells without cisplatin served as the control. For individual experiments, CM was pre‐incubated with anti‐human GDF15 neutralizing antibody.

For stimulation, endothelial cells EA.hy926 were cultured in the medium supplemented with 100 ng/mL GDF15.

### Quantitative reverse transcription‐polymerase chain reaction

Total cellular RNA was isolated using Trizol (Invitrogen, Carlsbad, CA, USA), and the reverse transcription reaction was conducted using RevertAid First Strand cDNA Synthesis Kit (Thermo Fisher Scientific, Waltham, MA, USA) according to the manufacturer's instructions. The specific primers were listed as follows: MMP9, 5′‐TCTTCCCCTTCACTTTCCTG‐3′ (sense), 5′‐CCCACTTCTTGTCGCTGTC‐3′ (antisense); VEGF, 5′‐CCAACTTCTGGGCTGTTCTC‐3′ (sense), 5′‐CCCCTCTCCTCTTCCTTCTC‐3′ (antisense); β‐actin, 5′‐CATGTACGTTGCTATCCAGGC‐3′ (sense), 5′‐CTCCTTAATGTCACGCACGAT‐3′ (antisense). PCR amplification was performed by FastStart Universal Probe Master (Roche, Basel, Switzerland). Relative quantification was analyzed by $2^{-\Delta \Delta C_{T}}$ method, and the expression levels of target genes were normalized to those of β‐actin.

### Western blots

Cells were lysed in RIPA buffer added with PMSF and phosphatase inhibitor (Beyotime Biotechnology, Jiangsu, China). Protein concentration was measured using the BCA method (Pierce, Rockford, IL, USA). Protein samples (10 μg) were separated by 10% SDS‐PAGE and transferred to polyvinylidene fluoride membranes (PVDF; Millipore, Billerica, MA, USA). After blocking with a buffer containing 5% low‐fat milk, the membrane was incubated with primary antibodies at 4°C overnight and the corresponding HRP‐conjugated secondary antibodies for 1 h at room temperature on the next day. Finally, the blots were developed with an enhanced chemiluminescence substrate (ECL; Pierce, Rockford, IL, USA). The phosphorylated protein was normalized to the corresponding total protein.

### Proliferation analysis

The proliferation of endothelial cells EA.hy926 was measured using WST‐1 cell proliferation assay (Roche, Basel, Switzerland) and 5‐ethynyl‐2′‐deoxyuridine (EdU) assay (RiboBIO, Guangzhou, China) according to the manufacturer's instructions. For WST‐1 assay, endothelial cells EA.hy926 were seeded into 96‐well plates at a density of 2 × 10^3^ cells per well and incubated with 100 ng/mL GDF15 for another 1–3 days. At the different time point, WST‐1 (10 μL) was added to each well and the absorbance at 450 nm was measured using a microplate reader (Thermo Fisher Scientific, Waltham, MA, USA). EdU assay was performed at day 3 to assess the proliferative ability of endothelial cells EA.hy926. After incubated with Edu working solution for 2 h, cells were stained with Hoechst solution. Pictures were captured in five randomly selected fields. Experiments were performed in triplicate.

### Tube formation in vitro

Endothelial cells EA.hy926 were seeded into 24‐well plates coated using Matrigel Matrix (BD Biosciences, San Jose, CA, USA) with or without 100 ng/mL GDF15 (4 × 10^4^ cells/well) and incubated at 37°C in an atmosphere of 5% CO_2_ for 4 h. The images were captured using a phase‐contrast microscope equipped with a digital camera in three random fields (100× magnification). The number of tubes was quantified by National Institutes of Health (NIH, Bethesda, Maryland, USA) Image software.

### Migration assay

A transwell migration system was used to study cell migration. Serum‐starved endothelial cells EA.hy926 seeded into the top chambers (6 × 10^4^/well) migrated to the bottom chamber where GDF15 (100 ng/mL) was added to medium or not. After 24 h, the cells on the upper surface of the membrane were removed and the lower surface were fixed with 4% paraformaldehyde and stained using 0.2% crystal violet. Migrated cells were counted from five random fields on each membrane at 100× magnification.

### Immunofluorescence

Cells were permeabilized with 0.1% Triton X‐100 after fixed with 4% paraformaldehyde on the ice. The primary antibody against paxillin (1:100) and fluorescein‐conjugated secondary antibody at 1:1000 dilution was applied. DAPI was used to stain the nuclei. The images were analyzed with the use of an Olympus fluorescence microscope at 200× magnification.

### Immunohistochemical analysis

The tumor tissues were deparaffinized, hydrated, heat‐induced antigen retrieved, and incubated with the primary antibody against GDF15 or CD31 at the dilution of 1:100 and 1:400 overnight at 4°C. The biotinylated secondary antibodies were used next day, and diaminobenzidine (DAB) was chosen as the substrate with hematoxylin counterstain.

### Animal experiments

The protocols of animal experiments were approved by the ethics committee of Zhongshan Hospital of Fudan University, Shanghai, China. All animal experiments were carried out according to the guidelines of the Shanghai Medical Experimental Animal Care Commission.

After treated with cisplatin, HCC cells Huh7 (2 × 10^7^) with or without endothelial cells EA.hy926 (4 × 10^5^) were subcutaneously injected into the right flank of 5‐week‐old BALB/c nude mice (*n* = 3 per group). The nude mice inoculated subcutaneously with nontreated Huh7 cells plus endothelial cells EA.hy926 (4 × 10^5^) were used as another control group (*n* = 3). Three weeks later, animals were sacrificed and tumor xenografts were harvested for immunohistochemistry.

Twelve mice bearing the tumors generated from chemotherapy‐damaged HCC cells Huh7 (2 × 10^7^) with endothelial cells EA.hy926 (4 × 10^5^) were divided into two groups randomly and treated with either DMSO (control group, *n* = 6) or thalidomide (50 mg/kg, three times a week, *n* = 6) intraperitoneally. After 4 weeks, animals were sacrificed and tumors were collected for analysis.

### Statistical analysis

Statistical analyses were performed by SPSS 13.0 software (Chicago, IL). All data are expressed as mean ± SD. The comparison between two samples or among three groups was analyzed using unpaired Student's *t*‐test or one‐way ANOVA analysis. A two‐sided *P* value of <0.05 was considered statistically significant.

## Results

### Increased GDF15 expression in chemotherapy‐damage to HCC cells

**Figure 1:**
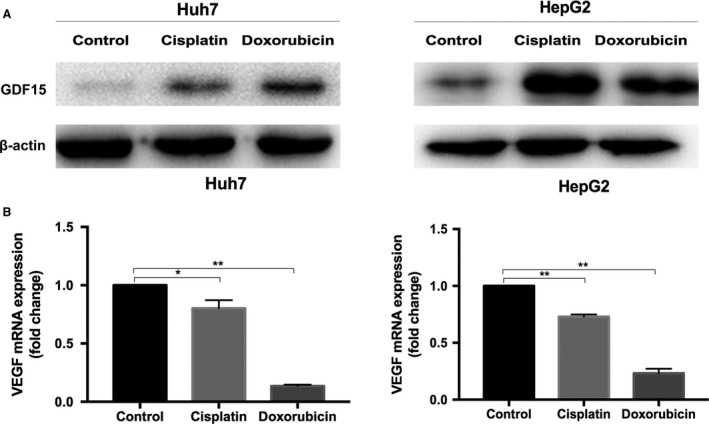
GDF15 was increased in the chemotherapy‐damaged HCC cells. (A) The protein expression of GDF15 was upregulated in the cisplatin‐ or doxorubicin‐treated HCC cells (Huh7 and HepG2) detected by Western blots. The β‐actin served as the loading control. (B) Quantitative RT‐PCR analyses showed the VEGF expression in cisplatin‐ or doxorubicin‐treated HCC cells (Huh7 and HepG2). *P <* *0.05, **P <* *0.01. HCC, hepatocellular carcinoma; GDF15, growth differentiation factor 15; VEGF, vascular endothelial growth factor.

We used the chemotherapeutic agent to treat HCC cells to mimic the chemotherapeutic damage of TACE to HCC. The GDF15 expression was significantly upregulated in HCC cells treated with cisplatin (Huh7, 6.9 μg/mL; HepG2, 65.2 μg/mL) or with doxorubicin (Huh7, 0.2 μg/mL; HepG2, 1 μg/mL) (Fig. 1A), whereas VEGF expression was markedly decreased (Fig. 1B). These data suggest that GDF15 not VEGF might be involved in the angiogenesis promoted by chemotherapy‐damaged HCC cells.

### In vitro angiogenesis enhanced by GDF15 from chemotherapy‐damaged HCC cells

**Figure 2:**
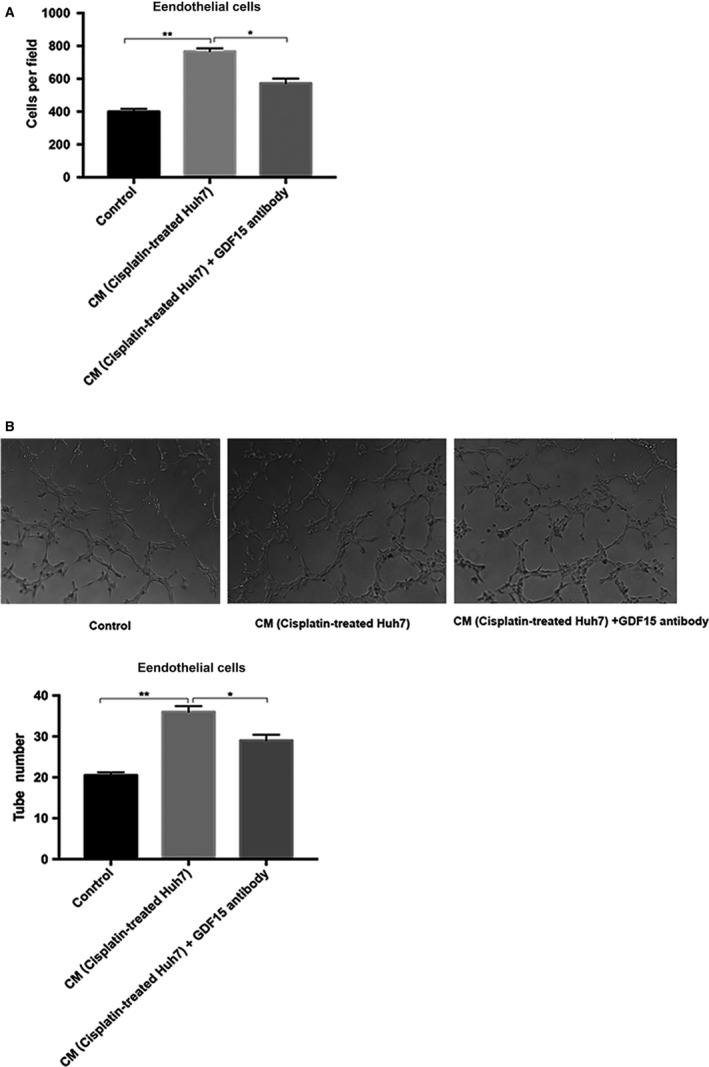
In vitro angiogenesis promoted by GDF15 from the chemotherapy‐treated HCC cells. (A) Migration of endothelial cells EA.hy926 was analyzed by Transwell assay (100× magnification). Serum‐starved endothelial cells EA.hy926 (6 × 10^4^ cell/well) were seeded into the upper chamber of Transwell, and the conditioned medium from nontreated Huh7 cells or cisplatin‐treated Huh7 was added to the bottom chamber. The anti‐human GDF15 neutralizing antibody was added into the conditioned medium from cisplatin‐treated Huh7. (B) Tube formation of endothelial cells. Endothelial cells EA.hy926 (4 × 10^4^ cells/well) were seeded 24‐well plates coated with Matrigel matrix in the presence of conditioned medium of nontreated Huh7, or cisplatin‐treated Huh7. The anti‐human GDF15 neutralizing antibody was added into the conditioned medium from cisplatin‐treated Huh7. The images were captured (100× magnification) from three random fields at 4 h. *P <* *0.05, **P <* *0.01. HCC, hepatocellular carcinoma; GDF15, growth differentiation factor 15.

Next, we investigated whether the chemotherapy‐damaged HCC cells increased angiogenesis through the release of GDF15. As shown in Figure 2, compared with the control medium, CM from chemotherapy‐damaged HCC cells significantly promoted migration and tube formation of endothelial cells EA.hy926. These effects could be substantially offset by adding anti‐human GDF15 neutralizing antibody into CM. These results indicate that chemotherapy‐damaged HCC cells promote angiogenesis through GDF15 secretion.

**Figure 3:**
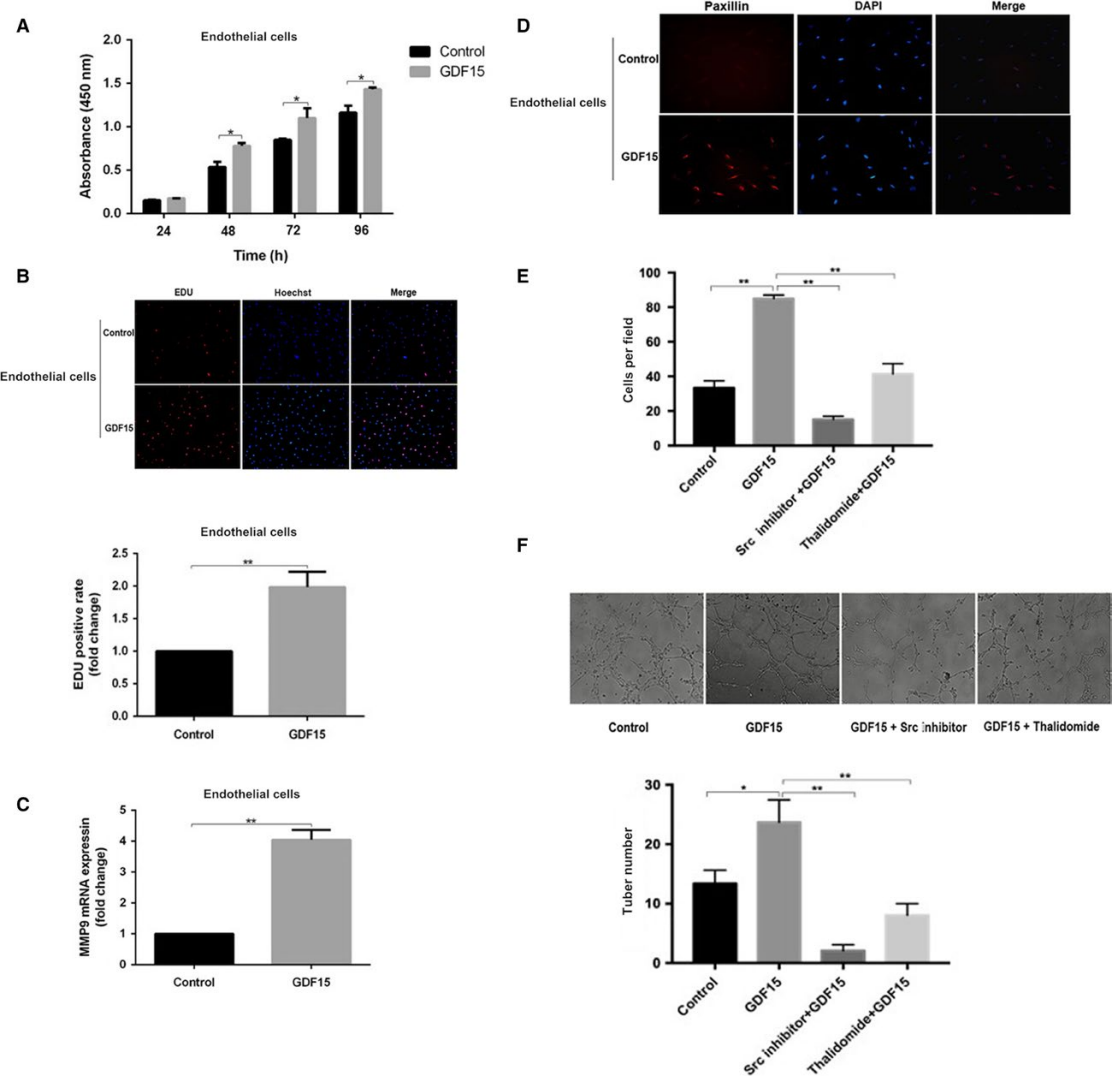
GDF15 promoted the proliferation, migration, and tube formation of endothelial cells. (A, B) The proliferation of endothelial cells EA.hy926 was detected by WST‐1 and EdU assays (200× magnification). (C) Quantitative analyses of MMP9 expression in the endothelial cells EA.hy926 treated with GDF15. (D) Paxillin expression in GDF15‐treated endothelial cells EA.hy926 was analyzed by immunofluorescence. Paxillin staining (red) and nuclei counterstained with DIPA (blue) (200× magnification). (E) Migration of endothelial cells was blocked by Src inhibitor or thalidomide. Endothelial cells EA.hy926 (6 × 10^4^ cells/well) were seeded into the upper chamber; control medium, GDF15 (100 ng/mL), GDF15 (100 ng/mL) with Src inhibitor (Saracatinib) or GDF15 (100 ng/ml) with thalidomide (100 μg/mL) was added to the bottom chamber. After 24 h, migration of endothelial cells EA.hy926 was analyzed by Transwell assay (100× magnification). (F) Tube formation of endothelial cells. Endothelial cells EA.hy926 (4 × 10^4^ cells/well) were seeded 24‐well plates coated with Matrigel matrix in the presence of control medium, GDF15 (100 ng/mL), GDF15 (100 ng/mL) with Src inhibitor (Saracatinib) or GDF15 (100 ng/mL) with thalidomide (100 μg/mL). The images were captured (100× magnification) from three random fields at 4 h. *P <* *0.05, **P <* *0.01. GDF15, growth differentiation factor 15.

To further assess the roles of GDF15 in angiogenesis, we used recombinant human GDF15 to stimulate endothelial cells EA.hy926. The process of angiogenesis involves basement membrane degradation, proliferation, migration of endothelial cells, and remodeling of new blood vessels. When compared with the control, GDF15 significantly promoted the proliferation, cell migration, and tube formation of endothelial cells EA.hy926 (Fig. 3A,B,E, and F). Migration‐related protein paxillin and matrix metalloproteinases MMP9 were markedly increased in GDF15‐stimulated endothelial cells (Fig. 3C and D), which are closely associated with migration of endothelial cells and degradation of extracellular matrix.

### Pro‐angiogenic effect of GDF15 through the activation of Src, AKT, MAPK, and NF‐κB signaling

**Figure 4:**
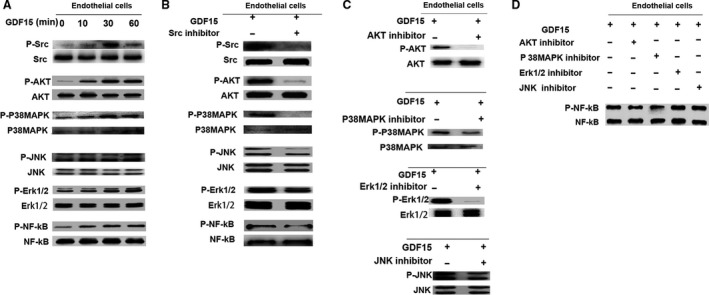
Pro‐angiogenic effects of GDF15 through the activation of Src, AKT, MAPK, and NF‐κB signaling (A) The phosphorylation of Src, AKT, P38MAPK, JNK, Erk1/2, and NF‐κB activation was detected by Western blots in the GDF15‐treated endothelial cells EA.hy926. (B) Pretreatment with Src inhibitor Saracatinib (2.7 nmol/L), the phosphorylation of Src, AKT, P38MAPK, JNK, Erk1/2, and NF‐κB was detected by Western blots in the GDF15‐treated endothelial cells EA.hy926. (C, D) Pretreatment with inhibitors of AKT (LY294002) (50 μmol/L), P38MAPK (SB203580) (10 μmol/L), JNK (SP600125) (20 μmol/L), or Erk1/2 (PD98059) (50 μmol/L) in the GDF15‐treated endothelial cells EA.hy926, activation of NF‐κB was examined by western blots. GDF15, growth differentiation factor 15.

Activation of Tyrosine kinase Src is required for angiogenesis 9. We next determined whether Src activation was involved in GDF15‐induced angiogenesis. When endothelial cells EA.hy926 were treated with recombinant GDF15, the phosphorylation of Src was significantly increased (Fig. 4A). The time profile of the phosphorylation of Src signaling after GDF15 treatment showed that the level of Src phosphorylation at the 60‐min time point was less than that at 30‐min time point. This suggests that Src phosphorylation in response to GDF15 was peaked at 30 min and then significantly decreased. In parallel with Src activation, GDF15 induced the phosphorylation of MAPK (Erk1/2, P38MAPK, JNK), AKT, and NF‐κB signal pathways (Fig. 4A). Pretreatment with Src inhibitor Saracatinib markedly diminished the cell migration and tube formation of GDF15‐treated endothelial cells EA.hy926 (Fig. 3E, and F). Meanwhile, the phosphorylation levels of MAPK (Erk1/2, P38MAPK, JNK), AKT and NF‐κB were significantly decreased (Fig. 4B). In addition, when GDF15‐stimulated endothelial cells were, respectively, pretreated with AKT or P38MAPK, ERK1/2 or JNK inhibitor, the corresponding GDF15‐trigged Src downstream signaling was significantly blocked (Fig. 4C). In parallel, the NF‐κB phosphorylation was significantly reduced when the cells were pretreated with AKT or P38MAPK inhibitor, whereas ERK1/2 or JNK inhibitor did not affect the NF‐κB phosphorylation (Fig. 4D). These data suggest that pro‐angiogenic effect of GDF15 is through the activation of Src and its downstream AKT, MAPK, and NF‐κB signaling.

### In vivo angiogenesis induced by chemotherapy‐damaged HCC cells

To determine whether chemotherapy‐treated HCC cells could promote the in vivo angiogenesis, chemotherapy‐treated HCC cells Huh7 mixed with or without endothelial cells EA.hy926 were subcutaneously injected into the nude mice. The expression of GDF15 and CD31 (a specific marker of endothelial cells) was significantly increased in the group of chemotherapy‐treated HCC cells mixed with endothelial cells EA.hy926 compared with the other two control groups (Fig. 6B). The tumor size of nontreated Huh7 cells mixed with endothelial cells was larger than the two groups of chemotherapy‐treated Huh7 cells with or without endothelial cells (Fig. 6A). These results show that chemotherapy‐damaged HCC cells promote in vivo angiogenesis via GDF15.

### Thalidomide suppressed the in vivo chemotherapy‐damaged HCC cells‐promoted angiogenesis

Thalidomide is an agent with anti‐angiogenic activity 10. We evaluated whether thalidomide could suppress the angiogenesis induced by the chemotherapy‐damaged HCC cells.

**Figure 5:**
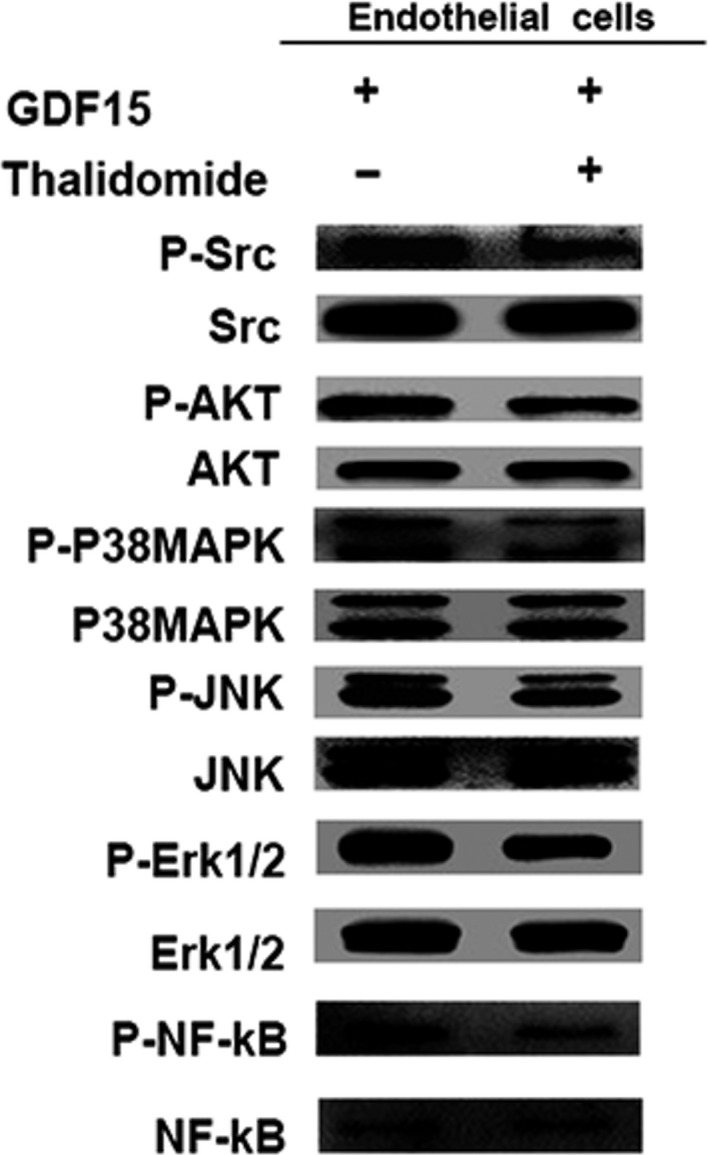
Thalidomide inhibited the GDF15‐induced activation of Src and its downstream pathways. Src, AKT, P38MAPK, JNK, Erk1/2, and NF‐κB were detected by Western blots in the GDF15‐treated endothelial cells EA.hy926 in the presence of thalidomide. GDF15, growth differentiation factor 15.

As shown in Figure 3E and F, thalidomide significantly inhibited migration and tube formation of GDF15‐stimulated endothelial cells EA.hy926. In terms of mechanism, the phosphorylation of Src and its downstream molecules induced by GDF15 was significantly decreased following the treatment of thalidomide (Fig. 5). These data indicate that thalidomide inhibits the in vitro angiogenesis induced by GDF15.

**Figure 6:**
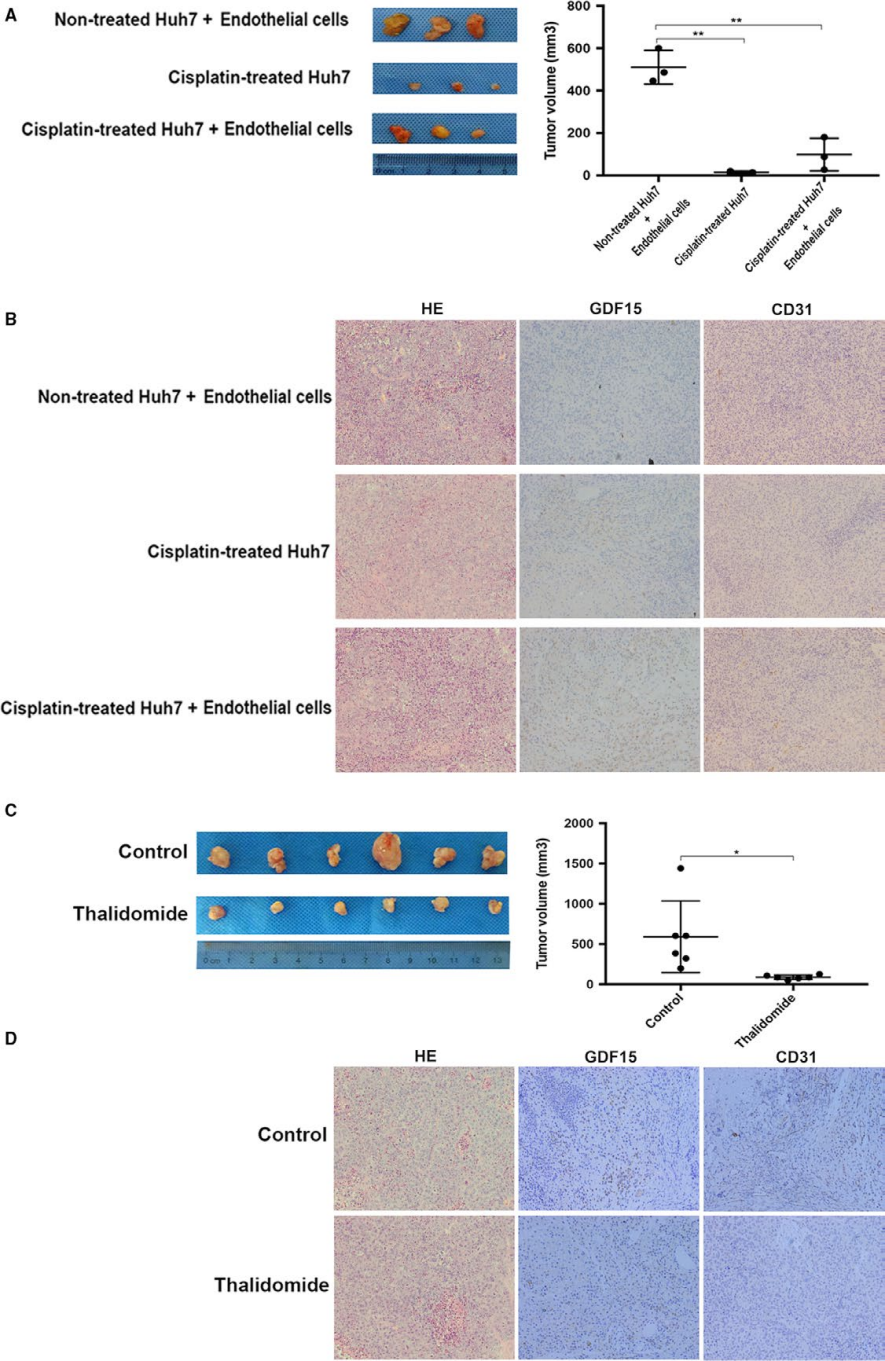
Thalidomide suppressed the in vivo chemotherapy‐damaged HCC cells‐promoted angiogenesis. (A) The tumor size in the group of cisplatin‐treated Huh7 with endothelial cells EA.hy926 was larger than that of cisplatin‐treated Huh7 alone, but significantly smaller than that of nontreated Huh7 with endothelial cells EA.hy926. (B) In vivo angiogenesis stained by CD31 was significantly increased in the group of cisplatin‐treated Huh7 with endothelial cells EA.hy926 when compared with two control groups. In parallel with increased angiogenesis, GDF15 was increased significantly (200× magnification). (C) The tumor size in the thalidomide‐treated group was significantly decreased. (D) Compared with the control group, a significant reduction in CD31 was observed after thalidomide treatment, but GDF15 expression was not significantly different. *P <* *0.05, **P <* *0.01. HCC, hepatocellular carcinoma; GDF15, growth differentiation factor 15.

Next, we investigated whether thalidomide could thwart the in vivo angiogenesis induced by chemotherapy‐damaged HCC cells. Mice bearing the tumors generated from chemotherapy‐exposed Huh7 with endothelial cells EA.hy926 were treated with thalidomide. The tumor size and angiogenesis indicated by the expression CD31 in thalidomide‐treated group were significantly decreased compared with the control group (Fig. 6C and D). The level of GDF15 expression did not differ significantly between the two groups.

## Discussion

Transarterial chemoembolization is the mainstay treatment for unresectable HCC; however, it provides a modest survival benefit in those patients 2. Among the factors interfering with its effectiveness, a theoretical neo‐angiogenic rebounce due to hypoxic induced by TACE is well known 11. To our knowledge, we firstly demonstrate that chemotherapeutic damage of TACE to HCC promotes tumor angiogenesis through the release of GDF15. Moreover, we propose thalidomide as a potential drug to target this detrimental pro‐angiogenic process. This study suggests that TACE combined with thalidomide could improve the treatment efficacy via disrupting the tumor angiogenesis induced by chemotherapeutic damage to HCC.

In previous studies, hypoxia‐induced VEGF increase by TACE was closely associated with treatment failure and poor prognosis of patients 3, 12. Chemotherapy is the essential component of TACE. In this study, we found that a new molecule GDF15 induced by chemotherapy damage to HCC is involved in tumor angiogenesis after TACE. This result adds a new insight into the mechanism of angiogenic rebound following TACE.

Growth differentiation factor 15 is a cellular stress‐related protein produced under various conditions including treatment 7, 13, 14. In this study, GDF15 was significantly upregulated in chemotherapy‐damaged HCC cells and promoted the migration and tube formation of endothelial cells to increase angiogenesis. Moreover, it increased the migration of endothelial cells and degradation of extracellular matrix via upregulating the expression of paxillin and MMP9.

Similar to other angiogenic factors, GDF15 induced the pro‐angiogenic effects through the phosphorylation of Src and its downstream pathways of AKT, MAPK, and NF‐κB signaling that are reported to be the important pathways in regulation of angiogenesis 15, 16, 17, 18. Thalidomide has anti‐inflammatory, immunomodulatory, and anti‐angiogenic activities 19, 20, 21. In this study, we demonstrated that thalidomide could suppress the in vitro and in vivo chemotherapy‐damaged HCC‐induced angiogenesis through targeting GDF15‐induced activation of Src and its downstream pathways. It has been reported that combined TACE with thalidomide can improve the survival of HCC patients 22. The present study maybe partially explains why combined with thalidomide can improve the treatment effectiveness of TACE.

**Figure 7:**
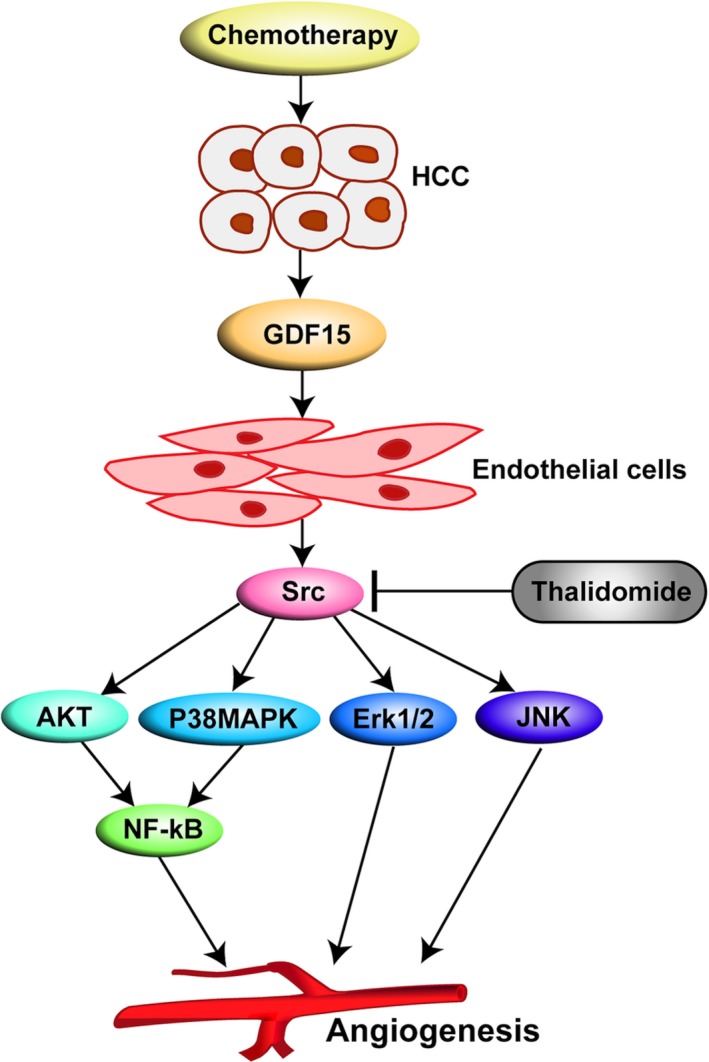
A graph shows that growth differentiation factor 15 (GDF15) from chemotherapy‐damaged hepatocellular carcinoma (HCC) cells promotes tumor angiogenesis and thalidomide reverses this pro‐angiogenic effect.

In conclusion (Fig. 7), we demonstrate that chemotherapeutic damage of TACE to HCC could promote tumor angiogenesis via the increased release of GDF15. Thalidomide could reverse these pro‐angiogenic effects, suggesting that thalidomide combined with TACE may improve the outcome of TACE.

## Conflict of Interest

The authors have no conflict of interests to declare.
